# Impact of sequence in concurrent training on physical activity, body composition, and fitness in obese young males: A 12-week randomized controlled trial

**DOI:** 10.1016/j.jesf.2025.02.001

**Published:** 2025-02-08

**Authors:** Zhen Li, Tingjun Gong, Ziyi Ren, Jian Li, Qinlong Zhang, Jinxi Zhang, Xiaohong Chen, Zhixiong Zhou

**Affiliations:** a Provincial University Key Laboratory of Sport and Health Science, School of Physical Education and Sport Science, Fujian Normal University, Fuzhou, 350117, China; b Capital University of Physical Education and Sports, Beijing, 100091, China

**Keywords:** Concurrent training, Body composition, Obesity, Young males

## Abstract

**Objectives:**

This study examined how different sequences of concurrent training impacted physical activity (PA), body composition, and physical fitness in young obese males. We also investigated whether the effectiveness of these interventions in reducing body fat percentage (BF%) was influenced by PA levels.

**Methods:**

A 12-week randomized controlled trial involving a cohort of 45 obese young males (mean age: 22.42 ± 1.96 years, mean BMI: 29.78 ± 3.37) was conducted. Participants were randomly assigned to three groups: the CRE group (Resistance Training (RT) followed by Endurance Training (ET)), the CER group (ET followed by RT), and the control group (Con). The training sessions were held three times a week. Measurements, including PA level, body composition, bone density, VO2max, and muscle strength, were assessed before and after the intervention.

**Results:**

Compared to those at baseline, following the intervention, both the CRE and CER groups showed significant improvements in various parameters, including PA level, body composition, bone density, VO_2max_, and muscle strength (*p* < 0.05), whereas no significant changes were observed in the Con group (*p* > 0.05). Specifically, the CRE group demonstrated remarkable progress, as evidenced by an increase in MVPA level (η^2^_p_ = 0.37, *p* < 0.001), a reduction in fat mass (η^2^_p_ = 0.28, *p* < 0.001), BF% (η^2^_p_ = 0.28, *p* < 0.001), android fat (%) (η^2^_p_ = 0.21, *p* < 0.001), gynoid fat (%) (η^2^_p_ = 0.30, *p* < 0.001), and various physical fitness indices, such as maximum strength (η^2^_p_ = 0.20, *p* = 0.008), explosive strength (η^2^_p_ = 0.38, *p* < 0.001), and muscular endurance (η^2^_p_ = 0.55, *p* < 0.001), surpassing the improvements observed in the CER and Con groups. Changes in PA levels during the intervention influence the efficacy of CT in reducing BF%.

**Conclusion:**

CT, particularly when RT precedes ET, had the potential to improve PA levels, overall physical fitness, body composition, and bone health in obese young males. Moreover, changes in PA levels during the intervention impacted the effectiveness of CT in reducing BF%.

**Trial registration:**

ChiCTR, ChiCTR2200063892.

## 1 Introduction

Since its classification as a disease in 1948, the prevalence of obesity has steadily increased.^1^ According to the World Health Organization (WHO), obesity is characterized by "an abnormal or excessive accumulation of fat that may pose a health risk," prompting a call for decisive measures to address this issue.^2^ Over the past two decades, there has been a rapid increase in obesity rates in developing countries, with the latest data indicating a continued increase in the prevalence of obesity.^2^^,^^3^ By 2030, it is estimated that the global population suffering from obesity will exceed one billion.^4^ Obesity not only impacts daily life but also serves as a primary factor in numerous chronic diseases (e.g., diabetes, musculoskeletal disorders, cardiovascular diseases, etc.) and specific types of cancer (e.g., gastroesophageal, breast, endometrial, ovarian, renal, and colon).^5^ Consequently, the ongoing increase in the prevalence of overweight and obesity has emerged as a critical public health issue.

Regular physical activity is widely regarded as a highly effective nonpharmacological intervention for the management of obesity, with a multitude of studies exploring diverse exercise intervention strategies, such as aerobic endurance exercise (ET) and resistance training (RT).^6^^,^^7^ ET, which encompasses activities such as running, swimming, and cycling, is widely recognized as an effective means for promoting weight loss and enhancing cardiovascular fitness, whereas RT primarily focuses on the enhancement of muscle strength and improvement of posture,^7^^,^^8^ Furthermore, for individuals with obesity, the augmentation of muscle mass through RT is of paramount importance, as it not only contributes to the improvement of body composition by reducing adipose tissue and increasing skeletal muscle mass but also enhances metabolic health, functional capacity, and overall quality of life.^9^ The guidelines established by the World Health Organization (WHO) emphasize the importance of regular engagement in both ET and RT, while the American College of Sports Medicine (ACSM) recommends moderate to high-intensity ET or RT to preserve or improve bone density.^10^^,^^11^ As previously noted, the integration of ET and RT, referred to as concurrent training (CT), appears to be one of the most effective therapeutic strategies for the obese population.^12^ Nonetheless, significant controversy and gaps persist in the understanding of the effects on body composition, bone mineral density, and muscle strength among obese young males, as well as the concurrent effects of ET and RT.

While numerous studies have focused on the superior efficacy of CT compared to that of individual exercise interventions in enhancing body composition among individuals with obesity,^13^, ^14^, ^15^, ^16^ it is notable that these investigations typically employ bioelectrical impedance analysis as the method of choice for assessing body composition. This method, which was originally designed for determining body fat percentage (BFP) in individuals with normal weight, may introduce inaccuracies when applied to obese individuals due to its underlying assumption of constant body hydration levels, thereby engendering potential measurement errors.^17^ Conversely, factors associated with CT, such as the order of exercise modalities, could introduce a confounding influence on the enhancement of muscle strength, thereby impacting the efficacy of exercise interventions in individuals with obesity.^18^, ^19^, ^20^ Moreover, the impact of CT on bone mineral density (BMD) remains unclear, given the well-documented association between obesity and diminished BMD and bone mineral content (BMC).^11^^,^^21^ Finally, previous investigations have overlooked the potential influence of intrinsic biological regulation on habitual physical activity (PA) or energy expenditure management over time.^22^ The ActivityStat hypothesis posits that alterations in PA within one domain may trigger compensatory adjustments in the opposite direction within another domain,^23^^,^^24^ thereby raising questions regarding whether CT interventions in obese populations might lead to reductions in PA levels across other domains. This knowledge gap hinders a comprehensive understanding of the mechanisms underlying the beneficial effects of exercise on obesity.

The primary objective of this investigation was to systematically compare the impacts of concurrent aerobic and strength training sequences on physical activity, body composition, and physical fitness among obese youth via a randomized controlled trial. In contrast to prior research,^13^^,^^14^ the novelty of this investigation lies in the implementation of a precise monitoring instrument, namely, a sports watch, for real-time monitoring of subjects' PA and for evaluating the interaction between PA and CT on subjects' body fat percentage (BF%). The strength of this methodology lies in its capacity to provide a more objective understanding of the repercussions of diverse training sequences on the holistic physical state of obese youth and to provide a more scientific basis for tailored obesity management.

## 2 Methods

### 2.1 Participants

The sample size was estimated utilizing G Power 3.1. According to a prior investigation,^16^ the test efficacy (1-β) was established at 0.80, while the incidence of type I error α was set at 0.05. Furthermore, the correlation between pre- and postintervention values was 0.80, with an effect size of 1.14. The necessary sample size per group was 12 individuals. In anticipation of a potential dropout rate of 20 %, it was decided that each group should comprise a minimum of 15 subjects, thus ensuring that the aggregate number of subjects would not fall below 45.

Forty-five young men, characterized by overweight and obesity, with an average age of 22.42 ± 1.96 years and a BMI of 29.78 ± 3.37, were enrolled in the present study. The recruitment of participants was conducted through various methods, including promotional posters displayed across universities in Beijing, as well as outreach through university health programs and social media platforms targeting potential subjects.

The inclusion criteria were as follows: 1) male aged between 18 and 30 years and exhibiting overweight conditions; 2) initial screening based on BMI; subsequently, body fat percentage was assessed using dual-energy X-ray absorptiometry, with a standard for obesity defined as male body fat percentage ≥25 %^25^; 3) no medical history of severe physical ailments or cardiovascular disorders such as hypertension or cardiac ailments; 4) no regular or concurrent physical training within the preceding six months; 5) maintenance of a relatively consistent lifestyle and dietary patterns throughout the trial, without significant alterations; 6) the capacity to comprehend and adhere to the research protocol, as well as complete the designated training regime and dietary assessments within the study timeframe; and 7) the ability to undergo all pertinent body measurements and laboratory examinations. The exclusion criteria were as follows: 1) factors that may influence the study outcomes, such as substance abuse, including alcohol or drugs; and 2) a prior history of weight reduction surgeries or other surgical procedures before enrollment.

Moreover, the researchers meticulously evaluated all prospective subjects against these standards, employing both the Physical Activity Readiness Questionnaire (PAR-Q) and the Medical Health/Medical History Questionnaire. To mitigate dropout rates, participants were informed that upon successful completion of the 12-week intervention characterized by high adherence, they would qualify to obtain a checklist for physical health assessment and a sports watch. Before commencement of the assessments, investigators elucidated the evaluation protocols, research aims, potential advantages, and drawbacks and furnished the subjects with informed consent documents for endorsement. Ethical approval was obtained from the Capital University of Physical Education and Sports Ethics Committee (code: 2022A48, approval date: 2022/09/12), and the study was registered in the Chinese Clinical Trial Registry (ChiCTR2200063892).

### 2.2 Study Design

**Fig. 1:**
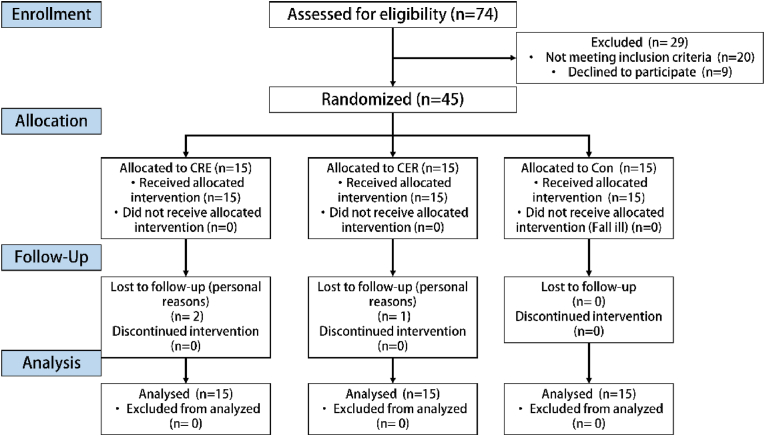
Flowchart of the participant selection process.

This investigation employed a randomized controlled trial (RCT) design, delineating participant eligibility and allocation processes, as illustrated in Fig. 1. The randomization of participants was conducted using SPSS software to generate random numbers, ensuring an unbiased allocation to the groups.Before the initial measurements, comprehensive explanations of all tests and training methods were provided to the participants. In total, 45 participants were randomly assigned to three groups: the concurrent resistance-endurance (CRE) group (n = 15), the concurrent endurance-resistance (CER) group (n = 15), and the control (Con) group (n = 15), as delineated in Table 1. Throughout the 12-week intervention period, no instances of injury were reported; however, three participants withdrew from the study for personal reasons—two from the CRE group and one from the CER group.

**Table 1:** Baseline demographic characteristics of the participants (n = 45).

Indicators (Units)	All (n = 45)	CRE (n = 15)	CER (n = 15)	Con (n = 15)
Age (years)	22.42 ± 1.96	22.20 ± 2.75	22.20 ± 1.15	22.87 ± 1.68
Height (cm)	176.06 ± 5.05	174.31 ± 4.36	176.33 ± 4.42	177.53 ± 5.99
Weight (kg)	92.49 ± 11.98	91.58 ± 13.42	92.31 ± 12.52	93.59 ± 10.58
BMI (kg/m^2^)	29.78 ± 3.37	30.08 ± 3.75	29.66 ± 3.61	29.61 ± 2.90
Body fat (%)	32.29 ± 4.80	32.07 ± 4.91	33.31 ± 5.34	31.49 ± 4.23

The investigation comprised a multidisciplinary team, encompassing assistants tasked with data collection, athletic trainers overseeing training sessions, and researchers overseeing training supervision and statistical analysis. The data were collected at three specific time points—baseline (September 2023), midterm (October 2023), and endpoint (January 2024)—with a postintervention assessment conducted 72 h after the conclusion of the training period.

### 2.3 Description of the intervention

In the preparatory phase preceding the commencement of intervention data collection, each participant underwent a comprehensive one-week concurrent training (CT) regimen, encompassing three distinct workout sessions meticulously designed to acquaint them with the principles of ET and RT. The primary objectives of this phase encompassed providing comprehensive instruction on appropriate weightlifting techniques, fostering familiarity with all exercise modalities and equipment, and ensuring uniform proficiency levels among all participants at the outset of the study.^26^

Table 2 delineates the contemporary training methodology implemented in this investigation, derived from the cyclic training regimen outlined in the ACSM guidelines.^27^ The week preceding the commencement of training was designated for the familiarization and assessment of maximal muscle strength (1-RM). Each training session was structured into three distinct components: (1) warm-up, (2) concurrent training, and (3) stretching. The duration of each training session spanned 60 min. Strength training was executed in alternating segments, encompassing a repertoire of exercises comprising bench press, deadlift, deep squat, shoulder abduction, plantar flexion, and bicep curls. Throughout the training sessions, escalating intensity levels (ranging from 50 % to 80 % of 1-RM) were employed alongside a reduction in repetitions for all exercises, except for abdominal and plantar flexion exercises. Bicep curls and plantar flexion exercises were performed without external resistance, maintaining a fixed repetition count of 15. The endurance training sessions were standardized to last for 30 min. Training intensity was incrementally elevated from 55 % of the maximal heart rate during the initial week to 75 % during the final week of the intervention. Exercise intensity was quantified utilizing a heart rate monitor (Polar S810, Polar Electro, Kempele, Finland).

**Table 2:** Organization of the 12-week concurrent training program.

weeks	1–3	4–6	7–9	10–12
Resistance Training				
Number of sets	3	3	3	3
Intensity (%1-RM)	15	12	10	8
Recovery time (s)	50	60	70	75
Endurance Training				
Exercise volume/time (min)	30	30	30	30
Intensity (HRmax)	50	60	70	75
Borg Scale	12	13	14	15

The CRE group diligently participated in the exercise regimen delineated in the RT and ET protocols within a single training session, where RT was systematically executed before ET. These sessions were rigorously conducted three times weekly, with each session meticulously scheduled for 60 min. Likewise, the CER group adhered to an analogous regimen, executing the exercise program within identical training sessions, where ET was administered prior to RT. These sessions were also conducted three times weekly, and each session was meticulously scheduled for 60 min. In contrast, the Con group underwent no intervention and maintained their regular daily activities. The Con group plays a vital role in this study by helping to eliminate confounding factors, validating the experimental process, interpreting results, and supporting scientific reasoning.

### 2.4 Study measurements

#### 2.4.1 Procedure

Body composition assessments were conducted at three distinct time points: preintervention, midintervention (specifically at week 4), and postintervention. Muscle strength and cardiorespiratory fitness were evaluated both pre- and postintervention. Furthermore, participants' PA levels were monitored throughout the intervention, with a particular focus on daily PA during the initial and final two weeks, which were meticulously recorded for subsequent analysis alongside dietary intake.

#### 2.4.2 Body composition analysis

Body weight was assessed using an electronic scale (Aimson Electronics Co., Ltd., Guangzhou, China). BMI was computed by dividing the body weight by the square of the height. Body composition and bone mineral density were assessed utilizing dual-energy X-ray absorptiometry (Lunar iDXA V17 software, GE Healthcare). The outcome variables included fat-free mass, fat mass, percentage of body fat (BF%), android fat (%), gynoid fat (%), and bone mass for the entire body (BMC and BMD). Measurements were conducted in a temperature-controlled environment following the guidelines provided by the manufacturer. All analyses were conducted by the same investigator to mitigate interobserver variability. The coefficients of variation for repeated measurements in our laboratory were 0.2 % for fat-free volume, 1 % for fat volume, and 0.2 % for bone mass of the entire body.

#### 2.4.3 Estimated VO_2_max

The multistage 20-m shuttle run test, employed for the estimation of VO_2max_, was implemented in this study. Participants were instructed to shuttle back and forth along a 20-m track, guided by audio signals, until they achieved a state of exhaustion.^28^ This test has been shown to be effective in estimating VO_2max_ in obese adults, a demographic that frequently encounters difficulties in participating in traditional laboratory-based assessments due to lower levels of PA and potential mobility constraints.^29^^,^^30^ The 20-m shuttle run test presents a practical and accessible alternative, enabling individuals to exert maximal effort within a familiar environment.

#### 2.4.4 Strength tests

Measurement of maximal leg strength: Isometric (single-joint) and isokinetic (multiple-joint) maximal leg strength was assessed using an IsoMed 2000 dynamometer (D&R Ferstl GmbH, Hemau, Germany) operating at a sampling rate of 200 Hz. Prior to each testing session, participants engaged in a structured warm-up comprising 10 min of supervised cycling on a stationary ergometer (Heinz Kettler GmbH and Co. KG, Ense-Parsit, Germany) at a submaximal workload of 0.75 W/kg body mass, maintaining a cadence of approximately 70 rpm.

Assessment of maximal isometric leg strength: Maximal isometric leg strength of the knee extensor and flexor muscles was evaluated with participants in a seated position, maintaining a backrest angle of 105° (where 180° corresponds to full extension), utilizing an IsoMed dynamometer equipped with a unilateral knee attachment. To minimize extraneous body movements during testing, straps were securely fastened across the shoulders, pelvis, and mid-thigh region. Additionally, participants were instructed to grasp the handgrips for further upper-body stabilization. The mechanical axis of the dynamometer was precisely aligned with the knee's axis of rotation, with the shin pad of the dynamometer lever arm positioned approximately 2–3 cm superior to the lateral malleolus. Gravity adjustment was meticulously conducted using the integrated software. Each testing session comprised two preliminary submaximal trials followed by three maximal trials under the following conditions: (a) right leg knee extension; (b) right leg knee flexion; and (c and d) identical sequence for the left leg. All assessments were conducted with the knee maintained at a 120° angle, with a 2-min rest interval between each trial.^31^^,^^32^

#### 2.4.5 Dietary assessment

Throughout the 12-week intervention, both prior to its commencement, during its execution, and subsequent to its conclusion, participants were tasked with diligently maintaining a detailed 3-day food diary aimed at discerning any potential disparities in dietary intake throughout the study period.^33^ Participants were instructed to meticulously document their dietary intake for two consecutive weekdays and one weekend day. Subsequent analysis of energy and macronutrient intake was conducted utilizing the Boohee Health software platform,^34^ accessible at https://www.boohee.com/apps/boohee.

#### 2.4.6 Physical activity assessment

**Fig. 2:**
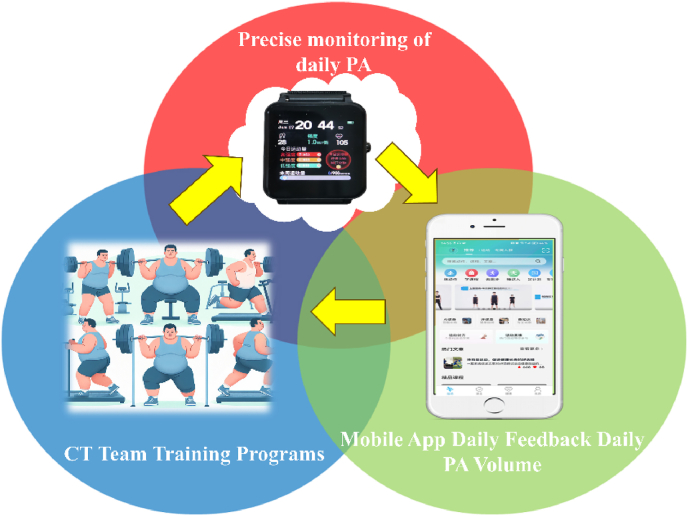
Physical activity assessment Note: Subsequent to the intervention group's completion of the prescribed CT program, daily PA was diligently monitored in real time via the SPORTS WATCH and systematically uploaded to the SHOUTI Fitness App, which consequently furnished the subjects with daily PA feedback.

The measurement of PA was conducted using a custom sports watch developed by researchers, which utilizes artificial neural networks for enhanced accuracy.^35^ This enabled nearly real-time continuous monitoring of PA levels. The device was capable of measuring not only the number of steps but also unique parameters quantifying the intensity of physical activity. Our device is based on data collected from a three-axis accelerometer, and the data is further optimized through neural network algorithms, which significantly improve the predictive validity of the measurements. The algorithm demonstrated an average absolute percentage error of 12.03 % and a mean squared error of 1.027, indicating high reliability and validity. Furthermore, we required participants to refrain from engaging in any RT during their daily PA outside of the intervention program.To monitor sedentary behavior and ensure consistency, we also ensured that participants maintained a regular daily routine, with similar class schedules and activity patterns.Data pertaining to PA were additionally captured via the "SHOUTI Fitness App," which established a daily feedback mechanism to deliver timely insights acquired from the application to the participants (Fig. 2).^36^

### 2.5 Statistical analysis

In this study, we utilized intention-to-treat (ITT) principles to handle missing values by employing multiple imputation (MI) methods and last observation carried forward (LOCF). Both methods provided consistent results.The acquired data were analyzed using IBM SPSS Statistics 26 software. Descriptive statistics, including the mean ± SD, were used to summarize the data. The Shapiro‒Wilk test was used to evaluate the normal distribution of variables, and the Levene test was used to test the homogeneity of variances. A repeated measures analysis of variance (ANOVA) was conducted with a 3 (group: CRE, CER, and Con) × 3 (time: pre, mid, and posttest) factorial design to evaluate the effects of the intervention before and after implementation for the experimental groups. Mixed linear models were employed to examine the intervention effects between groups, with the dependent variable being the rate of change in the pre- and posttests of the outcome indicator, while age and baseline values served as covariates. Dependent variables showing significance were further modeled with fixed effects for the null model of training (Con = 3; CRE = 2, CER = 1). The rate of change in PA, along with an interaction term, was subsequently incorporated into the fixed effects. Individual differences were treated as random effects. Furthermore, the effect size with statistical significance was calculated, expressed as η^2^_p_ and Cohen's d, where a small effect was defined as 0.01/0.20, a medium effect was defined as 0.06/0.50, and a large effect was defined as 0.14/0.80. The significance level was set at p < 0.05.

## 3 Results

### 3.1 Daily energy intake and levels of physical activity

No statistically significant differences (*p* > 0.05) were observed in the mean energy consumed among the groups throughout the trial period, as detailed in Table 3. Protein, carbohydrate, and fat constituted approximately 24 %, 60 %, and 16 %, respectively, of the daily energy intake across all groups. These proportions were consistent both before and after the intervention period.

**Table 3:** Assessment of participants' dietary intake and physical activity was conducted both before and after the 12-week intervention (mean ± sd).

Variables	Con		CRE		CER		Time effect η^2^_p_ (*P* value)	Group × time interaction η^2^_p_ (*P* value)
	Pre	Post	Pre	Post	Pre	Post		
Energy intake (kcal/d)	1843.87 ± 151.45	1848.20 ± 191.06	1846.47± 75.77	1923.60 ± 202.31	1827.80 ± 191.48	1741.87 ± 202.45	0.001 (0.970)	0.064 (0.248)
Energy intake (kcal/d/kg BW)	29.66 ± 3.31	28.08 ± 3.04	28.24 ± 2.89	28.59 ± 2.98	28.54 ± 4.34	28.57 ± 3.49	0.007 (0.577)	0.032 (0.500)
Protein (g)	93.84 ± 13.91	89.19 ± 11.73	89.07 ± 12.43	90.44 ± 11.53	93.75 ± 10.99	96.20 ± 12.25	0.001 (0.896)	0.051 (0.337)
Carbohydrate (g)	235.56 ± 25.52	255.29 ± 23.21	238.66 ± 26.23	258.41 ± 20.78	241.74 ± 28.78	262.18 ± 28.88	0.265 (<0.001)	0.001 (0.998)
Fat (g)	62.49 ± 8.49	60.49 ± 7.51	65.71 ± 9.46	60.93 ± 5.12	58.50 ± 9.26	62.84 ± 5.50	0.005 (0.646)	0.101 (0.108)
Saturated fat (g)	33.17 ± 5.71	33.25 ± 3.42	30.69 ± 3.40	33.07 ± 5.45	33.08 ± 4.37	32.84 ± 3.01	0.014 (0.452)	0.033 (0.492)
Unsaturated fat (g)	29.52 ± 3.53	31.00 ± 3.42	30.76 ± 3.75	31.57 ± 4.48	30.76 ± 3.46	31.47 ± 4.37	0.031 (0.253)	0.004 (0.925)
Steps	8487.53 ± 2093.01	8245.80 ± 1711.75	8086.60 ± 1330.96	11575.13 ± 1972.79	8275.01 ± 1455.56	9855.40 ± 2240.89	0.054 (<0.001)	0.512 (<0.001)
MVPA (min)	26.35 ± 8.66	25.31 ± 8.53	22.13 ± 5.58	42.85 ± 7.04	31.39 ± 8.67	39.33 ± 7.64	0.381 (<0.001)	0.367 (<0.001)

steps: Average daily steps; MVPA: moderate to vigorous physical activity.

With respect to PA volume, a notable interaction effect between group and time emerged for step (F_(2,45)_ = 22.055, *p* < 0.001, η^2^_p_ = 0.12) and MVPA (F_(2,45)_ = 42.816, p < 0.001, η^2^_p_ = 0.671). The CRE group (difference (95 % CI): 3488.53 (2686.97, 4290.10)), as well as the CER group (difference (95 % CI): 1580.40 (778.84, 2381.96)), manifested a significant increase in daily steps (p < 0.05). Similarly, both the CRE group (difference (95 % CI): 19.06 (16.12, 22.01)) and the CER group (difference (95 % CI): 11.86 (8.92, 14.81)) displayed a noteworthy increase in MVPA (p < 0.05). Between-group comparisons revealed that both the CRE and CER cohorts surpassed the Con group in augmenting daily step counts and MVPA (p < 0.05); moreover, the CRE cohort exhibited superior enhancements in MVPA compared to the CER cohort (p < 0.05).

### 3.2 Estimated VO_2_max and strength tests

The current investigation scrutinized the impact of the intervention on the estimation of VO_2_max and strength assessment. The outcomes of the investigation are depicted in Table 4, wherein time, group, and interaction exhibited statistically significant influences on the outcomes (*p* < 0.05). The intervention led to significant improvements in VO_2_max, maximum strength, explosive strength, and muscular endurance in both the CRE and CER groups (*p* < 0.05), with notably superior enhancement compared to that in the Con group (*p* < 0.05). Moreover, the CRE group outperformed the CER group in enhancing explosive strength and muscular endurance, with a notably superior effect compared to the Con group (*p* < 0.05).

**Table 4:** Changes in the estimated VO_2_max and strength tests before and after the intervention.

Variables	Group	Pre	Post	Δ%(95%Cl)	Cohen's d	ANOVA p value (η_p_^2^)	
						Time	Group × time interaction
VO_2_max (mL/kg/min)	Con	28.19 ± 3.43	28.37 ± 3.15	1.40 (−5.43,8.24)	–	<0.001 (0.522)	<0.001 (0.326)
	CRE	30.46 ± 3.94	34.39 ± 3.75	13.30 (10.13,16.48)∗	1.02		
	CER	28.39 ± 3.73	31.63 ± 2.97	11.59 (7.65,15.47)∗	0.97		
Maximum strength	Con	3.06 ± 0.40	3.09 ± 0.37	1.24 (−8.49,13.36)	–	<0.001 (0.702)	<0.001 (0.538)
	CRE	3.13 ± 0.35	3.78 ± 0.29	21.78 (14.08,29.47)∗	2.03		
	CER	2.81 ± 0.40	3.21 ± 0.35	15.03 (11.38,18.68)∗	1.07		
Explosive Strength	Con	2.53 ± 0.28	2.53 ± 0.26	0.07 (−2.12,2.26)	–	<0.001 (0.713)	<0.001 (0.586)
	CRE	2.53 ± 0.28	3.20 ± 0.18	28.23 (18.19,38.27)∗#	2.24		
	CER	2.64 ± 0.22	3.08 ± 0.22	17.21 (12.41,22.02)∗	1.47		
Muscular endurance	Con	0.69 ± 0.07	0.69 ± 0.05	−0.33 (−3.71,3.03)	–	<0.001 (0.900)	<0.001 (0.849)
	CRE	0.71 ± 0.09	0.90 ± 0.08	26.69 (23.53,29.85)∗#	2.24		
	CER	0.70 ± 0.05	0.81 ± 0.10	17.03 (14.82,19.25)∗	1.47		

Note: ∗ indicates a significant difference compared with the Con group (*p* < 0.05); # indicates a significant difference between the CRE group and the CER group (*p* < 0.05). Δ% represents the percentage change between pre- and posttests. Within-group comparisons were analyzed using repeated-measures ANOVA; between-group comparisons were analyzed using analysis of mixed linear models (controlling for pretest data and age).

### 3.3 Body composition and bone densitometry

**Fig. 3:**
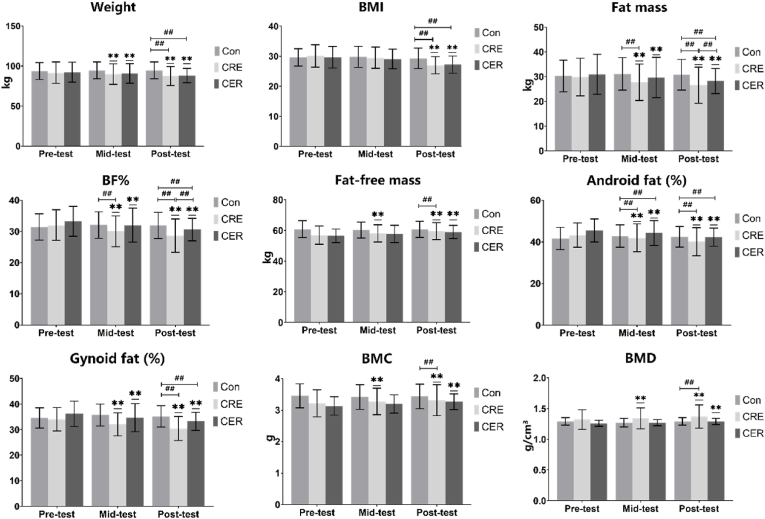
Changes in body composition and bone mineral density after 12 weeks of intervention ∗ denotes a noteworthy contrast for intragroup comparisons (*p* < 0.05); # signifies a notable difference for intergroup comparisons (*p* < 0.05).

The results of the repeated-measures ANOVA indicated significant effects for Weight (F_(2,45)_ = 5.73, *p* < 0.001, η^2^_p_ = 0.214), BMI (F_(2,45)_ = 5.17, *p* < 0.001, η^2^_p_ = 0.197), Fat mass (F_(2,45)_ = 7.949, *p* < 0.001, η^2^_p_ = 0.275), BF% (F_(2,45)_ = 8.256, *p* < 0.001, η^2^_p_ = 0.282), Fat-free mass (F_(2,45)_ = 8.313, *p* < 0.001, η^2^_p_ = 0.284), Android fat (%) (F_(2,45)_ = 5.641, *p* < 0.001, η^2^_p_ = 0.212), Gynoid fat (%) (F_(2,45)_ = 8.793, *p* < 0.001, η^2^_p_ = 0.295), BMC (F_(2,45)_ = 4.538, *p* = 0.002, η^2^_p_ = 0.178), and BMD (F_(2,45)_ = 4.630, *p* = 0.002, η^2^_p_ = 0.196). This was evidenced by a significant difference (p < 0.05) observed between the CRE and CER groups both before and after the intervention in various body composition indices, including weight, BMI, fat mass, BF%, fat-free mass, android fat (%), gynoid fat (%), and BMC and BMD indices. The application of mixed linear modeling revealed notable differences (p < 0.05) between the CRE and CER groups in weight, BMI, fat mass, BF%, fat-free mass, android fat (%), gynoid fat (%), BMC, and BMD compared to the Con group postintervention; furthermore, differences between the CRE and CER groups were observed in BF%, fat mass, and BMD (p < 0.05)(Fig. 3).

### 3.4 Effect of PA on the intervention effect

In the null model (Table 5), with body BF% serving as the dependent variable, both CRE and CER exhibited notable impacts (p < 0.001); upon integrating ΔSteps (Model 1), the impacts of CRE and CER remained significant (p < 0.001); upon further integration of ΔMVPA (Model 2), the impacts of CRE and MVPA were statistically significant (p < 0.05), whereas the impact of CER was nonsignificant (p > 0.05); and upon simultaneous integration of both ΔSteps and ΔMVPA (Model 3), neither the impacts of CRE nor CER were statistically significant, nor was the influence of PA (p > 0.05).

**Table 5:** Mixed linear model of BF% intervention effect.

Model	Parameters	Estimate	t	p	95%Cl	
					Lower	Upper
Null Model	CRE	−0.09	−8.17	<0.001	−0.18	−0.11
	CER	−0.09	−5.19	<0.001	−0.13	−0.06
Model 1	CRE	−0.15	−4.72	<0.001	−0.21	−0.09
	CER	−0.08	−3.77	<0.001	−0.13	−0.04
	ΔSteps	<-0.001	0.04	0.69	−0.001	0.002
	CRE × ΔSteps	<-0.001	−0.003	−2.29	−0.002	0.002
	CER × ΔSteps	<-0.001	−0.004	−2.48	−0.003	0.001
Model 2	CRE	−0.12	−4.21	<0.001	−0.18	−0.06
	CER	−0.05	−1.49	0.14	−0.12	0.02
	ΔMVPA	<0.001	2.16	0.04	−0.007	−0.001
	CRE × ΔMVPA	<-0.001	−0.58	0.56	−0.007	−0.0004
	CER × ΔMVPA	<-0.001	−0.16	0.87	−0.007	−0.001
Model 3	CRE	−0.09	−1.16	0.25	−0.25	0.07
	CER	−0.04	−0.61	0.54	−0.19	0.10
	ΔSteps	<0.001	0.82	0.42	−0.0001	0.002
	ΔMVPA	0.003	1.81	0.08	−0.0003	0.007
	CRE × ΔSteps × ΔMVPA	<0.001	−0.96	0.34	−0.0003	0.0001
	CER × ΔSteps × ΔMVPA	<0.001	−0.96	0.34	−0.0003	0.0001

## 4 Discussion

Prior research has established a significant association between PA and obesity. Given the limited data regarding the impact of CT on daily PA, the precise relationship between CT interventions and changes in body composition remains ambiguous.^37^ In this investigation, the implementation of a precision monitoring device facilitated real-time tracking of participants' daily PA, which was subsequently correlated with both ET and RT sequences. This innovative methodology not only yields comprehensive and precise data that enhances our understanding of how different training sequences affect the physical condition of obese young males, but also offers novel insights into the interplay between training order and PA levels. After a 12-week training intervention, both the CRE and CER groups demonstrated significant enhancements in daily PA, body composition, and critical physical fitness metrics, including muscle strength and cardiorespiratory fitness, relative to the control group. While the differences in VO_2max_ between the two forms of CT were minimal, the CRE group exhibited significantly greater improvements in muscle power and endurance compared to the CER group. In light of the escalating obesity epidemic and the insufficient levels of physical activity, this study presents a practical and effective strategy for enhancing daily PA and improving body composition among obese young males.

The current investigation initially evaluated the impacts of the two CT regimens on the daily energy intake and levels of PA among participants. Throughout the duration of the trial, it was noted that there was no notable variance in the mean daily energy intake among the groups, and the ratio of protein, carbohydrate, and fat within the daily energy intake remained consistent, thereby negating the dietary factor as a confounding variable in this research. Furthermore, this implies that the intervention did not have a noteworthy impact on energy intake, and participants managed to maintain a moderately stable dietary pattern throughout the trial, aligning with prior research outcomes.^11^ Furthermore, the present investigation revealed that in relation to physical activity metrics, including step count and MVPA, noticeable increases were observed in both the CRE and CER groups, while the control group showed minimal changes. Of particular significance is the clearly superior improvement in MVPA observed within the CRE cohort. This implies that CT does not lead to a decrease in PA in this population^23^; rather, it promotes daily PA, which offers an additional intervention option with practicality and feasibility for public health guidelines on PA. Common interventions employed in experimental studies aimed at promoting PA include sustained MVPA, setting goals for step counts, and providing educational guidance.^38^

The association between exercise intervention and daily PA has attracted significant scholarly attention. The current investigation demonstrated the effectiveness of CT in improving daily PA, including steps and MVPA, among obese male youth through precise monitoring of PA. Previous research has also investigated alternative exercise interventions, such as HIIT, using accelerometers to increase daily PA.^22^ These studies suggest that exercise interventions, by enhancing cardiorespiratory fitness (CRF), promote greater PA,^39^ supported by the observed increase in VO_2max_ among obese youth males in our study. However, a substantial body of literature has identified a decline in daily PA levels subsequent to engagement in exercise,^24^ termed the "ActivityStat hypothesis".^23^ For instance, Holliday et al. noted a statistically nonsignificant alteration in MVPA levels after a 24-week intervention comprising 150 min of moderate-intensity exercise weekly.^40^ Furthermore, apart from CRF serving as an influential factor, this study posits that the meticulous monitoring protocol employed herein (utilizing a sports watch to precisely monitor daily PA volume and furnishing daily feedback to participants via a mobile application) also contributes to the augmentation of daily PA. Additionally, personalized PA prompts have emerged as highly efficacious behavioral change techniques (BCTs) for augmenting individuals' daily PA volume in prior investigations.^41^ Additionally, the remarkable improvement in MVPA levels in the CRE group compared to the CER group may be attributed to improvements in muscular strength in the CRE group. Previous investigations have shown that RT not only improves muscular strength but also increases explosive power, thus promoting participants' ability and willingness to engage in moderate-to-vigorous physical activity.^42^ Of particular note is the clear advantage in muscular strength demonstrated by the CRE group compared to the CER group within the scope of this study.

Consistent with prior studies,^43^, ^44^, ^45^ the present investigation reaffirms that the sequencing of CT does not significantly affect the enhancement of VO_2max_, which is a critical indicator of cardiorespiratory fitness (CRF). CRF encompasses the ability of the circulatory and respiratory systems to supply oxygen to skeletal muscle mitochondria, thereby facilitating the production of essential energy for physical activities and serving as a crucial measure of physical vitality, psychological health, and academic performance in young individuals.^39^ Sale et al.^46^ demonstrated that the CT group exhibited no incompatible effects on mitochondrial density, citrate synthase (CS), or phosphofructokinase (PFK) activity in comparison to a standalone ET group. This assertion is further corroborated by Bell et al.,^47^ who found that the enzyme activity of succinate dehydrogenase (SDH) in type I and IIa muscle fibers was comparable between the CT and pure ET groups. In contrast, a decrease in enzyme activity was noted in strength-focused groups concerning muscle fiber mitochondrial density. Notably, the rate of increase in the CT cohort was analogous to that of the ET cohort, indicating that CT can effectively augment aerobic capacity without undermining the physiological adaptations linked to endurance training. Furthermore, the increases in SDH and CS enzyme activity, along with mitochondrial density—critical aerobic enzymes for evaluating muscle fiber endurance capacity—were consistent across both concurrent and endurance-focused training regimens.^48^^,^^49^ This compatibility establishes a physiological foundation for the absence of interference effects of CT on CRF.

Nonetheless, the arrangement of training sequences influences the muscular attributes of the participants. The current investigation revealed that the CT protocol incorporating RT prior to ET yields markedly superior outcomes compared to those of its counterpart, substantially augmenting explosive strength and muscular endurance, albeit leaving maximum strength unaffected. This recurring pattern has also been observed in antecedent research endeavors.^19^^,^^20^^,^^50^ The fundamental mechanism behind this interference phenomenon is yet to be fully understood; antecedent research has proposed that initiating ET as the initial regimen markedly diminishes the number of repetitions achievable during back squat exercise,^51^ consequently influencing the enhancement of muscular strength. However, it is noteworthy that the volume of RT training remained consistent across both intervention cohorts in the current investigation. Additionally, exercise sequencing could modulate the participants' readiness for training, potentially compromising their subjective perception of readiness due to the physiological strains induced by ET, thereby impacting subsequent RT sessions.^52^ Markov et al. provided evidence indicating that ET induces both central fatigue (impaired neural drive) and peripheral fatigue (impaired excitation–contraction coupling), culminating in a decrease in muscular strength.^53^ Furthermore, metabolic factors stimulated by ET emerge as pivotal contributors; for instance, ET precipitates a decrease in Ca^2+^ sensitivity, potentially impeding the translation of electrical stimuli into mechanical responses.^54^, ^55^, ^56^ Findings from both animal and human investigations illustrate that physical exertion (e.g., cycling at 70 % $\dot{V}$O_2max_) triggers heightened phosphorylation of ryanodine receptor 1, disrupting Ca^2+^ handling within myofibers and consequentially impacting muscle function.^57^ Concurrently, metabolic acidosis (e.g., diminished pH and/or Pi accumulation) and compromised action potentials (e.g., extracellular K+ buildup) seem to be exacerbated by ET.^57^ This body of evidence implies that acute disruptions primarily affect metabolic rather than neuromuscular attributes, consequently compromising training efficacy and impeding chronic adaptation.

It is widely agreed upon that CT surpasses a single exercise modality in enhancing body composition among individuals with obesity^18^; however, this investigation revealed greater efficacy in fat oxidation when RT precedes ET. Goto et al. ascertained that the levels of fatty acids, glycerol, and growth hormone in serum were elevated when RT preceded ET.^58^ Additionally, sequential RT and ET foster heightened levels of markers such as lipocalin and cortisol among participants, hormones pivotal in modulating energy metabolism and bolstering the catabolic environment, thereby culminating in weight reduction.^13^^,^^59^ Consequently, the heightened fat oxidation observed in the RT followed by ET (CRE) group may be attributed to the augmented lipolysis stemming from the antecedent performance of RT. Furthermore, Sheikholeslami-Vatani et al. corroborated this phenomenon^59^; nonetheless, the current investigation revealed a more substantial reduction in the percentage of fat in patients receiving RT followed by ET (CRE) in the Android group than in those receiving a concurrent exercise regimen (CER). Elevatedroid fat percentage typically correlates with increased susceptibility to metabolic and cardiovascular ailments.^60^ The propensity of abdominal fat to induce health complications surpasses that of fat accumulation in other regions, such as the hips and thighs.^61^ This phenomenon ensues from the heightened propensity of abdominal fat infiltration into the liver and other visceral organs, culminating in metabolic irregularities and heightened inflammatory responses. Moreover, elevatedroid fat percentage exhibits a robust correlation with increased susceptibility to ailments, including insulin resistance, diabetes, hypertension, and cardiovascular disorders.^62^ Hence, the findings of this study carry significant clinical ramifications.

Obesity intricately influences bone metabolism and protein synthesis, predominantly within myocytes, consequently frequently correlating with diminished BMD and BMC among individuals with obesity, thereby increasing the propensity for osteoporosis and fractures.^63^ Chronic inflammation and skeletal muscle atrophy are acknowledged as principal risk factors for osteoporosis.^64^ Proinflammatory cytokines, including TNF-α and C-reactive protein (CRP), play pivotal roles in bone resorption and osteoclast differentiation. Moreover, within the context of obesity, heightened leptin secretion and/or diminished lipocalin synthesis by adipocytes may directly or indirectly impinge upon bone formation via the upregulation of proinflammatory cytokine generation.^65^ Regular physical activity can potentially confer anti-inflammatory effects by mitigating visceral adiposity (through the attenuation of adipokine secretion) and by fostering an anti-inflammatory microenvironment postexercise.^66^ ET and RT are frequently employed to mitigate susceptibility to inflammation-related disorders; nevertheless, amalgamating both RT and ET, known as CT, within a unified training regimen may potentiate the anti-inflammatory properties of habitual physical activity, thereby exerting repercussions on bone metabolism.^13^ In the current investigation, both CT intervention modalities elicited an increase in BMC and BMD among obese males, with no discernible differences observed between them. Previous investigations revealed markedly elevated concentrations of lipocalin, CTRP5, CTRP9, CRP, and TNF-α in individuals who underwent CT with RT prior to ET, indicating a plausible influence of exercise sequence on the efficacy of CT on inflammatory markers.^13^ Given the prolonged duration required for bone remodeling processes and the 12-week intervention timeframe in this study,^67^ forthcoming investigations might extend the intervention duration to ascertain the impact of the CT sequence on bone metabolism.

Finally, given the potential for physiological compensatory responses within the body, such as diminished PA, heightened sedentariness, or elevated energy consumption, to sustain PA or energy equilibrium amid escalated levels of high-intensity PA, this emerges as a contributing factor to the incongruity observed across studies investigating exercise interventions.^23^ Furthermore, empirical investigations have demonstrated a correlation between PA and obesity, particularly with MVPA.^68^ Hence, incorporating daily PA as a covariate is deemed judicious in experimental inquiries wherein body composition serves as the dependent variable. The current investigation corroborated the presence of an impact stemming from alterations in PA levels on the efficacy of reducing BF%. Following a 12-week CT intervention, enhancements in both the participants' levels of PA and their body composition were observed. Subsequent modeling analyses revealed that incorporating PA as a predictor attenuated the impact of CT on ameliorating BF%. Remarkably, when simultaneously accounting for both CT and PA (Steps × MVPA), neither exhibited a noteworthy influence on BF%, possibly attributable to the robust correlation between CT intervention and the promotion of PA. This study demonstrated that the efficacy of CT in enhancing BF% was modulated by the level of PA. Subsequent research endeavors may delve deeper into discerning whether the impact of CT intervention on body composition is mediated by the facilitation of PA, with elucidating the role of exercise intensity and volume in shaping body composition serving as pivotal to this exploration.

### 4.1 Strengths and limitations

The present investigation demonstrated several noteworthy strengths. First, it employed an innovative approach to monitor participants' daily physical activity using advanced surveillance equipment, which allowed for precise correlations between these activities, ET, RT, and BF%. This innovation not only enhances our understanding of the physical condition of obese youth but also holds significant clinical implications for developing tailored interventions. Second, the rigorous randomized controlled trial design effectively minimized biases, thereby enhancing the credibility of the findings. Additionally, multiple indicators, such as body composition, physical activity level, and muscle strength, were considered, facilitating a comprehensive assessment of the effects of different training sequences on obese male youth. Finally, meticulous data collection methods ensured the accuracy and objectivity of the data, further bolstering the reliability of the study results.

Nevertheless, this study has limitations. First, the modest sample size may limit the generalizability of the conclusions, highlighting the need for future research with larger cohorts. Moreover, the evaluation of upper limb strength was omitted, although existing literature suggests that CT variables have a negligible impact on this measure. Dietary intake, being self-reported, introduced potential recall bias and variability; however, our analysis indicated no significant interaction effects between groups over time (p > 0.05), suggesting minimal influence from diet. Additionally, factors such as sedentary behavior, sleep patterns, and psychological stress were not controlled; however, given that participants were in the middle of the academic semester, these factors remained relatively stable and exhibited minimal variability. Lastly, the relatively short duration of the intervention may not fully capture the long-term effects of exercise on skeletal health. Future investigations should continue to explore the implications of CT variables on obesity while expanding the scope to include diverse age groups, genders, and body mass indices.

## 5 Conclusion

The execution of CT has the potential to enhance daily PA levels, fortify CRF, augment muscular strength, optimize body composition, and reinforce bone health among obese young males; furthermore, fluctuations in PA levels intricately influence the enhancement of BF% induced by CT. When aiming to maximize MVPA levels, enhance muscular explosiveness and endurance, and refine body composition, it is advisable to follow RT prior to engaging in ET pursuits.

**Table:** Abbreviations

WHO	World Health Organization
PA	Physical activity
ET	Aerobic endurance exercise
RT	Resistance training
CT	Concurrent training
CRE	Concurrent Resistance-Endurance
CER	Concurrent Endurance-Resistance
MI	Multiple imputation
LOCF	Last Observation Carried Forward
BFP	Body fat percentage
BMD	Bone mineral density
BMC	Bone mineral content
PAR-Q	Physical Activity Readiness Questionnaire
ACSM	American College of Sports Medicine
VO2max	Maximum Oxygen Uptake
ITT	intention analysis
MVPA	Moderate to Vigorous Physical Activity

## Author contributions

Zhen Li: Conceptualization, Methodology, Investigation, Writing - Original Draft, Writing - Review & Editing; Tingjun Gong: Methodology, Investigation, Data Curation, Writing - Review & Editing; Ziyi Ren: Investigation, Data Curation, Writing - Original Draft; Jian Li: Investigation, Data Curation, Visualization; Qinlong Zhang: Investigation, Resources, Writing - Review & Editing; Jinxi Zhang: Investigation, Data Curation, Visualization; Xiaohong Chen: Resources, Writing - Review & Editing; Zhixiong Zhou: Funding Acquisition, Conceptualization, Study Design, Development and Implementation of the Study Protocol, Drafting the Manuscript, Supervision, Project Administration, Writing - Review & Editing.

## Data availability statement

The datasets used in the present study are available from the corresponding author upon reasonable request.

## Ethics approval and consent to participate

Ethical approval was obtained from the Capital University of Physical Education and Sports Ethics Committee (code: 2022A48, approval date: 2022/09/12), and the study was registered in the Chinese Clinical Trial Registry (ChiCTR2200063892). Informed consent was obtained from the subjects prior to the start of the study.

## Funding

This work was funded by the 10.13039/501100012166National Key Research and Development Program of China (Grant No. 2020YFC2006200) and the Emerging Interdisciplinary Platform for Medicine and Engineering in Sports (EIPMES).

## Declaration of interest Statement

The authors declare that there are no conflicts of interest regarding the publication of this paper.
